# Cancer of Testicle

**Published:** 1871-02

**Authors:** T. G. Williams

**Affiliations:** Watertown, Wis.


### CANCER OF TESTICLE, ETC.

By T. G. WILLIAMS, M.D., of Watertown, Wis.

Lewis D., German, age 28, conductor on street-car, noticed, six months ago, an enlargement of his right testicle; consulted a physician, who ascribed it to gonorrhœa, from which he was then suffering. It was treated accordingly. Symptoms abated at first. Subsequently, there were exacerbations of pain and swelling; after four months it commenced growing steadily and somewhat rapidly, no treatment seeming to control it. Pain of a lancinating character, and, previous to admission, the weight was very troublesome.

Admitted to Mercy Hospital, March, 1868. Prof. Andrews diagnosed it malignant and advised its removal. It was removed in the usual manner.

The growth was solid, of a pearly white color, and fibro-cartilaginous in structure. Weight, 12 ounces.

Recovered from the operation in the usual time, and nothing of interest was noticed, except the petulance of the patient and some enlargement of the cord. This, subsequently, passed away.

Returned to his work about the first of May, apparently as well as ever. Saw him a month after. He appeared to be in perfect health and good spirits.

Readmitted, in the latter part of June, in an unsconcious and paralyzed condition. Evacuations involuntarily, staining his linen bright yellow. Bleeding considerably from a small wart on right shoulder; controlled only by a ligature about the base.

This and a small growth on the abdomen, to the right of the umbilicus, the size of a pea, were all that could be seen externally that was abnormal. Continued thus a few days, and, finally, died.

*Post mortem* examination revealed the cause of the symptoms. The base of the middle lobe of the right hemisphere of the brain was extensively disorganized, implicating the bones beneath it, and there were to be seen many other less extensive deposits in various parts of the same hemisphere. But a few were found in the left hemisphere. The right lung was almost completely degenerated into a dark mass. This solidification was very apparent upon physical examination before death. The right lobe of the liver was extensively implicated also. The kidney and liver were united together by a growth which had its origin from the pelvis of the kidney, and was in size equal to three times that of a healthy kidney. The substance of the kidney was but little changed. The walls of the right ventricle of the heart contained two small deposits. The left lung was but little implicated. The left lobe of the liver and the left side of the heart, and the left kidney, and the left testicle were not visably implicated.

These deposits were melanotic, differing from the original affection of the testicle, which was white. The interest connected with the case, is that the deposits were almost wholly in the right half of the body, and the suddenness of the onset of the fatal symptoms. Nothing unusual was noticed two weeks before his death.

*Query.* Was the final result hastened by interference in removing the testicle?

